# Developing In Situ Chemometric Models with Raman Spectroscopy for Monitoring an API Disproportionation with a Complex Polymorphic Landscape

**DOI:** 10.3390/ph16020327

**Published:** 2023-02-20

**Authors:** Shikhar Mohan, Yi Li, Kevin Chu, Bing Shi, Liliana De La Paz, Prarthana Bakre, Chris Foti, Victor Rucker, Chiajen Lai

**Affiliations:** 1 Gilead Sciences, Foster City, CA 94404, USA; 2 Velexi Corporation, Burlingame, CA 94010, USA; 3 Information Systems, Naveen Jindal School of Management, The University of Texas at Dallas, Richardson, TX 75080, USA

**Keywords:** Raman spectroscopy, X-ray diffraction, process analytical technology, process monitoring, salt disproportionation

## Abstract

An in situ Raman method was developed to characterize the disproportionation of two salts involving a complex polymorphic landscape comprising up to two metastable and one stable freebase forms. Few precedents exist for Raman calibration procedures for solid form quantitation involving more than two polymorphs, while no literature examples were found for cases with multiple metastable forms. Therefore, a new Raman calibration procedure was proposed by directly using disproportionation experiments to generate multiple calibration samples encompassing a range of polymorph ratios through in-line Raman measurements complemented by off-line reference X-ray diffraction measurements. The developed Raman methods were capable of accurately quantitating each solid form in situ when solid concentration variation was incorporated into the calibration dataset. The kinetic understanding of the thermodynamically driven polymorphic conversions gained from this Raman method guided the selection of the salt best suited for the delivery of the active ingredient in the drug product. This work provided a spectroscopic and mathematical approach for simultaneously quantitating multiple polymorphs from a complex mixture of solids with the objective of real-time monitoring.

## 1. Introduction

A majority of the drug candidates in research and development are classified as Biopharmaceutics Classification System (BCS) class II with high permeability but low aqueous solubility [1]. BCS class II drug substances are of great concern for drug delivery as low solubility often results in low bioavailability. Salt formation is the most preferred and cost-effective development strategy for increasing solubility [1,2,3,4,5,6,7,8,9]. However, an intrinsic liability of this approach is the natural tendency for the salt to revert to the less soluble unionized form via a proton transfer reaction, known as disproportionation. Previous studies [10,11,12] have reported cases where the unionized drug forms compromised the bioavailability [10], physical integrity [12], and/or stability [11] of the drug product. Therefore, the proportion of the salt and its counterpart free base or acid (unionized) forms is often considered as a critical quality attribute (CQA) for both the drug substance and drug product. With FDA’s initiative toward quality by design (QbD) [13], it is important to monitor and control this CQA with process analytical technology (PAT) to improve process understanding and ensure the final product quality meets the required specifications.

Raman spectroscopy is commonly used as a PAT tool in the pharmaceutical industry to monitor salt formation and disproportionation during drug substance and product development [14,15,16,17,18,19,20,21]. This spectroscopic tool is a light scattering technique that induces changes in molecular vibrations and rotational energies associated with the polarizability of the sample. This technique’s rapid measurement speed, flexibility and portability of instrumentation, nondestructive nature, and low sensitivity to water are particularly suitable for in-line measurement during batch reactions. The spectral range of Raman measurements is typically from 40 to 4000 cm^−1^. This range includes the chemical fingerprint region (400–4000 cm^−1^) and the low frequency/far infrared region (40–400 cm^−1^). Raman spectroscopy is ideal for quantitative monitoring of salt disproportionation since different salts, and free base polymorph forms have unique Raman signatures [19].

The development of a quantitative method requires a calibration procedure to address challenges associated with Raman spectroscopy, such as signal interferences and fluorescence. This was emphasized by Simone et al. [22], where the authors defined a ‘good calibration practice’ for developing a Raman method for monitoring polymorphic transformation in a crystallization process. This practice involved three steps: (1) identifying parameters that can change during a crystallization experiment, (2) verifying the sensitivity of Raman with the sample before experimentation, and (3) using design of experiments (DoE) to generate calibration samples for model development. Amongst the crystallization parameters studied by Simone et al., solid content had a strong effect on the Raman spectra. Therefore, once the sensitivity of polymorph forms of the studied substance (o-aminobenzoic acid (OABA) forms I and II) was confirmed, a DoE encompassing control for polymorph ratios and the solid content was used for model development. The most accurate model developed was based on a chemometrics technique, partial least squares (PLS), which deconvoluted and extracted the Raman signal associated with the form change. The chemometric model was successfully deployed to monitor OABA form II conversion to form I in a supersaturated solution. Similar calibration procedures for monitoring solid form conversion have been reported in the literature for both aqueous and tablet systems, including a recent study by Nie et al. [17] at Merck. The authors created both reflectance and transmission Raman models for monitoring pioglitazone hydrochloride salt (PIO-HCl) as it disproportionated to its free base form in tablets at different stress conditions (40 °C/75% RH and 40 °C/35% RH). Since this was a tablet system, the calibration samples were tablets with a controlled ratio of PIO-HCl to the free base form. These studies emphasized the importance of controlling crystallization parameters, such as the solid content in aqueous systems, through the preparation of calibrations samples. However, the complexity of calibration sample preparation with controlled polymorph ratios increases dramatically as more polymorphs are involved. Few precedents exist for model development strategies in cases with more than two polymorphs. This study seeks to apply an appropriate calibration procedure for monitoring salt disproportionation for an active pharmaceutical ingredient (API) referred to as compound X that had a complex polymorph landscape of up to three free base forms derived from the disproportionation of two salt forms.

The salt formation of compound X was recently studied to gain insight into crystallization kinetics [20]. The selection of a compound X salt for drug delivery required comparing the disproportionation rate constants of several compound X salts. Therefore, quantitative Raman models were developed in this study to monitor the quantity of each solid form during disproportionation. Developing Raman models for compound X salt formation and disproportionation encountered challenges not addressed by previous calibration procedures.

The primary challenge associated with compound X was the presence of multiple polymorphs of the free base. Three of the polymorphs were observed during polymorph screening; thus, four different solid forms (salt and three polymorphs of the freebase forms) might co-exist during disproportionation. The Raman spectra for these polymorphs were all highly similar, introducing tremendous challenges for spectral deconvolution. The co-existence of several solid forms also created concerns about controlling the polymorph ratios in calibration samples. As mentioned earlier, the DoEs reported in the literature for similar applications generally involved a control on the ratio of two solid forms either in an aqueous system or in tablets. However, it was not feasible in this work when dealing with more than two polymorphs that were limited in the amounts of materials and in the characteristics for differentiation by Raman. Additionally, the dynamic nature of the system, where metastable freebase forms either changed to other metastable forms or converted to the most stable freebase form, made controlling the polymorph ratio a challenge.

As compared to previously reported studies that used mixtures of well-characterized polymorphs, this study used in situ mixtures of the relevant polymorphs during the actual disproportionation. This procedure involved directly performing a disproportionation experiment and obtaining in-line Raman spectra and simultaneous offline sampling for X powder-ray diffraction (XRPD) measurements. The advantage of this approach was that multiple relevant polymorphs could be generated concurrently, allowing for the mathematical deconvolution of Raman spectra for accurate quantitation of the solid forms for future experiments. During this work, solid concentration and counterion effects were also investigated by performing additional disproportionation experiments at varying solid concentrations and with two different salts. The effort of Raman method development for compound X salts led to defining a new calibration approach, which pharmaceutical scientists can use for mechanistic assessment of a complicated polymorphic landscape to enhance drug quality control.

## 2. Materials and Methods

### 2.1. Materials

Two salts of the API, Compound X, were produced to monitor their disproportionation. The two salts differed in the counterion acid (i.e., hydrochloric acid (HCl) and maleic acid) and were referred to as HCl salt and maleate salt, accordingly. Up to three polymorphs of the free base (form I, form II, form V) of compound X were observed, and form V was the most stable one. Pure water without any pH modifiers was used as the solvent for this investigation.

### 2.2. Experimental Setup

**Figure 1:**
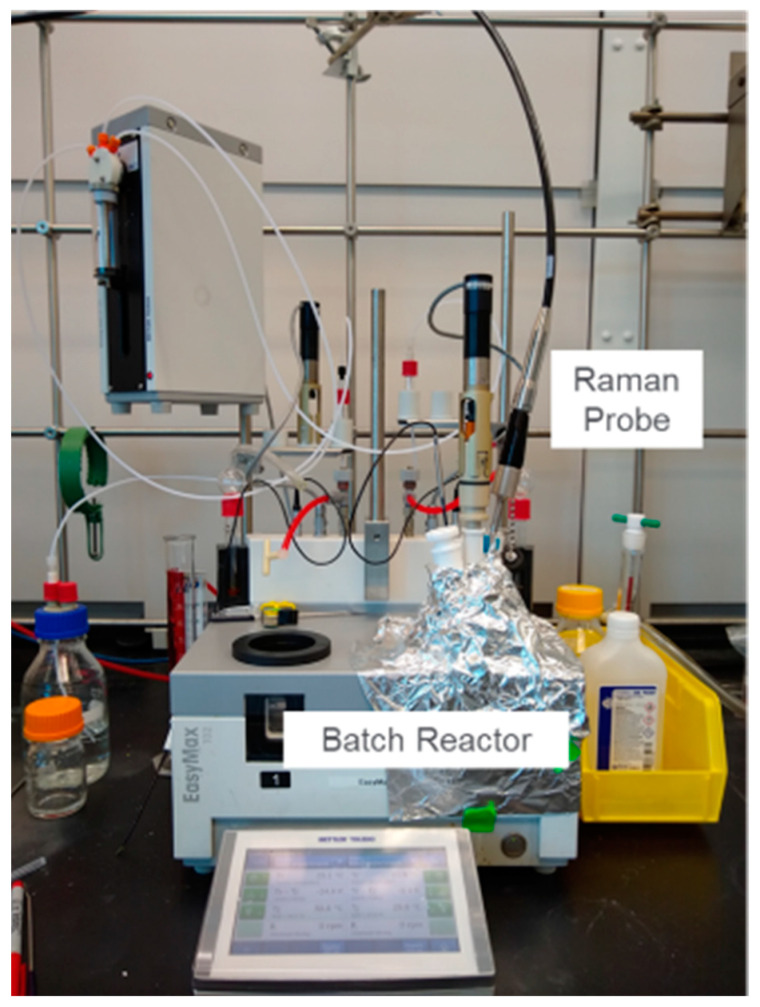
Experimental Setup.

The disproportionation experiments were performed in a lab-scale glass vessel (EasyMax model 402, Mettler-Toledo, Columbus, OH, USA) with concentrations ranging from 12.5 mg/mL to 50 mg/mL (Figure 1). The agitation and batch temperature were set at 500 rpm and 50 °C, respectively, throughout the study. An in-line Raman probe (Kaiser Optical Systems Inc., Ann Arbor, MI, USA) was placed in the vessel for process monitoring. The solid concentration was controlled at the start of each experiment. The salt of Compound X was weighed out and charged into approximately 100 mL of HPLC-grade water in the EasyMax vessel. During the study, four disproportionation experiments were performed for each salt resulting in a total of eight experiments. Table 1 shows the experiments performed and their respective solid concentration of either 12.5, 16.7, 25.0, or 50.0 mg/mL. In situ Raman spectra at every other minute and approximately 9–17 offline X-ray measurements spanning the entire disproportionation experiments were collected. All the experimental runs were monitored for approximately 450 min. Three sets of experiments were assigned as calibration runs for model development, and one set was assigned as test runs for model optimization and evaluation. The solid concentration of 16.7 mg/mL was chosen for the test run because this concentration was in the range of the concentrations used for all the experiments, and the concentration was high enough to produce Raman data with a high signal-to-noise ratio.

### 2.3. X-ray Diffraction

X-ray powder diffraction (XRPD) patterns were collected with a PANalytical X’Pert PRO MPD diffractometer using an incident beam of Cu Kα radiation produced using a long, fine-focus source and a nickel filter. The diffractometer was configured using the symmetric Bragg-Brentano geometry. Prior to the analysis, a silicon specimen (NIST SRM 640e) was analyzed to verify the observed position of the Si 111 peak was consistent with the NIST-certified position. A specimen of the sample was prepared as a thin, circular layer centered on a silicon zero-background substrate. Anti-scatter slits (SS) were used to minimize the background generated by air. Soller slits for the incident and diffracted beams were used to minimize broadening from axial divergence. Diffraction patterns were collected using a scanning position-sensitive detector (X’Celerator) located 240 mm from the sample and Data Collector software v. 2.2b.

During the disproportionation process, offline samples were collected periodically for X-ray diffraction measurements. Binary physical mixtures of various forms (e.g., HCl salt vs. form V, form I vs. form V) were prepared in 10% increments (from 0–100%) and subjected to X-ray diffraction measurements. At least two signature peaks of each form were selected for peak area integration, and a calibration curve (composition vs. integrated area) was established for each binary pair of forms. For the process samples, the fraction of each form was determined by finding the relative amount of all binary pairs and applying the assumption that the percentage of all forms in the process, including the salt and the freebase forms, added up to 100%. The quantitation results from X-ray diffraction were used as a reference in the development of chemometric Raman models.

### 2.4. Raman Spectroscopy

A Kaiser Raman RXN1 system with an F/1.8 imaging spectrograph with a HoloPlex transmission grating was utilized to acquire Raman spectra to enable in situ reaction monitoring. The fiber optics used a 250 mW, 785 nm laser for excitation and a TE-cooled 1024 CCD detector. The outputted Raman spectrum had a spectral range from 100 to 3425 cm^−1^ with a resolution of 1 cm^−1^. Optimized acquisition parameters of 60 s exposure time and 60 s delay between scans were used for all spectral collection. Data acquisition was performed through the icRaman software (Mettler-Toledo, Columbus, OH, USA), while chemometric modeling and spectral preprocessing were performed using MATLAB (version R2019b, Mathworks Inc., Natick, MA, USA) and PLS_Toolbox (version 821, Eigen-vector Research Inc., Wenatchee, WA, USA).

### 2.5. Methodology for Spectral Investigation of Solid Form, Solid Concentration, and Type of Salt Effect

The disproportionation of HCl or maleate salt was monitored using Raman spectroscopy. For each salt, a calibration dataset (see Section 2.6) was required to encompass the variation of process and output variables critical to the disproportionation. The critical variables investigated were the polymorph ratio and solid concentration.

The polymorph ratio was the most important variable to consider during calibration because it was the primary critical quality attribute of interest. During all the experimental runs, the solid forms might include HCl salt, maleate salt, freebase form I, freebase form II, and freebase form V. The Raman spectra of pure forms were obtained, and their peaks were compared. These Raman spectra, along with the rest of the process spectra, were truncated from 100–3425 cm^−1^ down to 100–1761 cm^−1^ to reduce noise and emphasize the form-specific regions.

In addition to the polymorph ratio, the solid concentration was also investigated. Disproportionation experiments were performed at four controlled solid concentrations of 12.5, 16.7, 25, and 50 mg/mL. Raman spectra collected at the end of disproportionation for each solid concentration and salt combination were compared for observed spectral variations. The Raman spectra were normalized with standard normal variate (SNV), which centered each spectrum at zero by subtracting Raman shift intensities from the mean of all intensities of a spectrum followed by dividing by the standard deviation of the intensities. This normalization helped to reduce the baseline effect and highlight the peak intensity changes.

Lastly, Raman spectra encompassing the variation due to the two salts were also compared. For spectral comparison, the Raman spectra collected at the end of disproportionation for only the experimental run with a solid concentration of 16.7 mg/mL were used. This allowed spectral comparison with fixed solid concentration. The solid concentration of 16.7 mg/mL was specifically chosen because it was the test run condition as defined in Section 2.2. Normalized (SNV) spectra were compared for observed spectral variations associated with the polymorph-specific Raman peaks.

A collective analysis of spectral variations associated with solid form, solid concentration, and salt was also performed using principal component analysis (PCA). The unique directions of spectral variability were extracted with PCA and plotted in a reduced dimensional space referred to as the scores plot. Additionally, the latent variables plots from PCA were used to extract information on the shape of the dominating variability. Only the Raman spectra associated with all calibration runs, as defined in Section 2.2, were used for the PCA analysis.

### 2.6. Calibration Procedure

Quantitative models for polymorph form fractions were developed with Raman spectroscopy using calibration run experiments. Calibration runs were disproportionation experiments, in which approximately 9–17 in situ Raman spectra of co-existing polymorphs, along with corresponding offline X-ray measurements spanning the entire duration of the process, were collected to generate the quantitative models. In total, there were three calibration runs varying in the solid concentration of 50.0 mg/mL, 25.0 mg/mL, and 12.5 mg/mL for each salt. Model sets were produced from the calibration run datasets using two calibration strategies: without and with concentration effect, defined in Section 2.6.1 and Section 2.6.2. Each model set consisted individual PLS models for the fraction of every polymorph present during the disproportionation. Specifically, a model set for HCl salt experiments included three PLS models of mass fractions for form I, form II, and form V. The fraction of HCl salt was calculated by subtracting all the fractions of the freebase polymorphs from unity. This approach was confirmed by XRPD since HCl salt reference values approximately matched the calculated values. The model set for maleate salt experiments was similar, except that there were no models developed for form I due to its absence in these experiments. The individual models were optimized and evaluated using root mean square error of prediction (RMSEP) on a test run with a solid concentration of 16.7 mg/mL.

An initial set of models were also developed and referred to as baseline models. These models were used as a benchmark to compare all other models since the model was generated using only the test run dataset. Raman spectral preprocessing for the baseline models involved truncation of spectral range to emphasize solid-form-indicating regions and mean centering to comply with the PLS algorithm. The baseline models were evaluated using cross-validation. The type of cross-validation used was a venetian blind with 10 splits, and the resulting root mean square error of cross-validation (RMSECV) was used to compare with the RMSEP of the calibration procedure models. Table 2 shows the calibration procedure model sets and the baseline models, which were compared independently for both salts. The calibration procedure associated with each model set are described in subsequent sections (Section 2.6.1 and Section 2.6.2).

#### 2.6.1. Calibration Procedure without Inclusion of Concentration Effect

For the calibration procedure without concentration effect (CP), only one calibration run conducted at a solid concentration different from the test run experiment was used to develop the models (Table 2). The model development followed the same procedure as the generation of the baseline model. This procedure was used to develop three individual sets of models (model sets 1–3) for each of the two salts. The calibration run condition associated with model sets 1–3 corresponds to solid concentrations of 50 mg/mL, 25 mg/mL, and 12.5 mg/mL, respectively. By changing the calibration run condition for these model sets, the effect of the solid concentration on the prediction performance was investigated. Chemometric models with partial least squares (PLS) were developed to deconvolute the spectra and reduce the effect of solid concentration. Spectral preprocessing included mean centering to comply with the PLS algorithm. The number of latent variables used for each model was individually optimized based on the minimization of (RMSEP).

#### 2.6.2. Calibration Procedure with Inclusion of Concentration Effect

The calibration procedure with concentration (CP_Conc_) utilized spectroscopic data from multiple solid concentrations to create one model set for each salt as opposed to creating multiple model sets for each solid concentration (Table 2). The advantage of combining the run conditions for calibration was that the solid concentration was incorporated in model development as opposed to solely relying on chemometric techniques to reduce the concentration effect. Spectral preprocessing used for the maleate salt experiments with this procedure was mean centering, while for the HCl salt experiments were SNV and autoscaling. The selection of preprocessing and the number of latent variables were based on the minimization of RMSEP.

## 3. Results and Discussion

### 3.1. Effect of Solid Form, Solid Concentration, and Type of Salt on Raman Spectra

**Figure 2:**
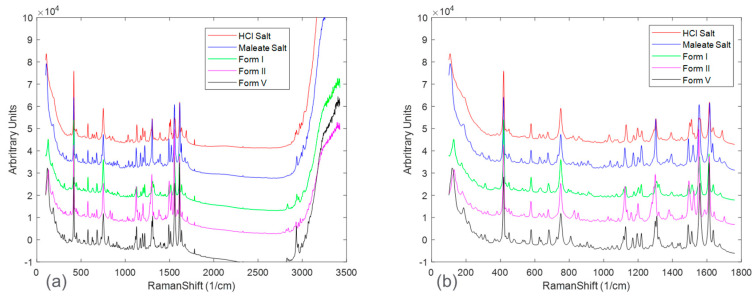
Full (**a**) and truncated (**b**) pure component Raman spectra (baseline offset for ease of visual comparison) of the two salts (HCl and Maleate) and the three observed freebase polymorphs.

The pure-form Raman spectrum for each solid form was collected for comparison and shown in Figure 2. Figure 2a shows the full spectra, in which, beyond the Raman shift of approximately 1800 cm^−1^, there was mostly baseline and noise with limited presence of sample-specific Raman peaks. Once the Raman spectra were appropriately truncated, spectral peaks associated with each solid form were able to be compared. However, fingerprint Raman peaks from each solid form were highly overlapping, presenting a challenge for quantitative modeling.

**Figure 3:**
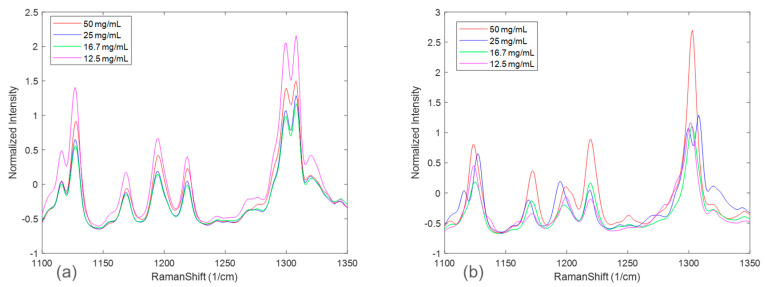
Raman spectra at the end of disproportionation monitoring for HCl salt (**a**) and maleate salt (**b**) color−coded based on solid concentration.

Figure 3 zooms in on Raman spectral peaks from four solid concentrations at the end of the disproportionation for both HCl and maleate salts. For the HCl salt, the spectral changes associated with solid concentrations were observed to be peak intensity offsets. The maleate salt, in addition, showed peak shifts corresponding to solid concentration variation. These effects could be explained by considering an expanded Beer-Lambert law where the Raman intensity is defined as a linear combination of each polymorph, the solute, and the solvent [23]. In a suspension system, particle size and solid concentration are multiplicative parameters in the expanded Beer-Lambert law and may potentially cause peak intensity height variations. Note that the increases in peak intensities were not completely rank-ordered with solid concentrations for both the salt systems. This phenomenon, in addition to the apparent peak shifts present in maleate salt, could be attributed to confounding interferences from solute/solvent concentration, fluorescence, and/or interactions amongst the variables, e.g., polymorph ratio and salt concentration.

**Figure 4:**
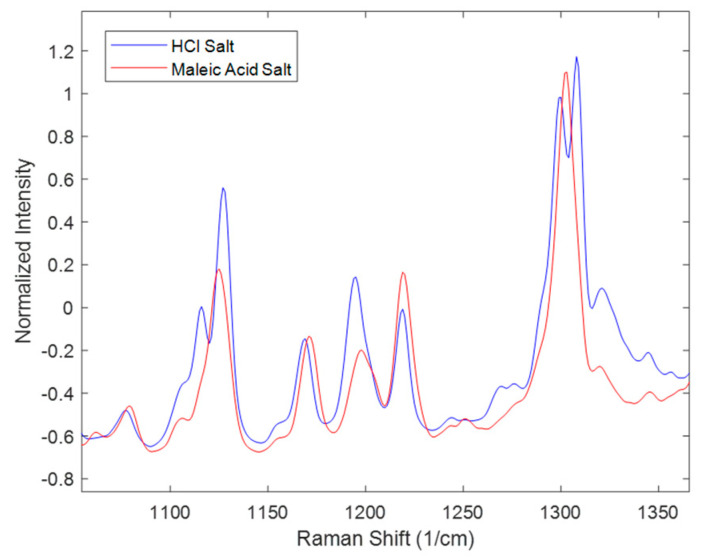
Raman spectra of the completed disproportionation process for HCl salt and maleate salt of the 16.7 mg/mL test run condition.

Similarly, spectral comparisons were performed for the effect of salt seen in Figure 4. The spectra in Figure 4 represent the 16.7 mg/mL solid concentration at the end of the disproportionation; thus, interpretations of this figure only refer to this condition. The Figure zooms in on peaks characteristic of the polymorphs. Not all the peaks match as in the range of 1100–1150 cm^−1^ and at around 1300 cm^−1^ where HCl salt has two peaks while maleate salt has one peak. In addition, in the range of 1150–1250 cm^−1^, there were differences in the peak height ratios of the three peaks. These differences were attributed to the presence of multiple polymorphs and their different ratios at the end of the disproportionation. Polymorph form I was not observed in the entire maleate salt disproportionation process; while it was present in most of the HCl salt processes. Additional interactions between the process parameters might also account for the observed spectral differences.

**Figure 5:**
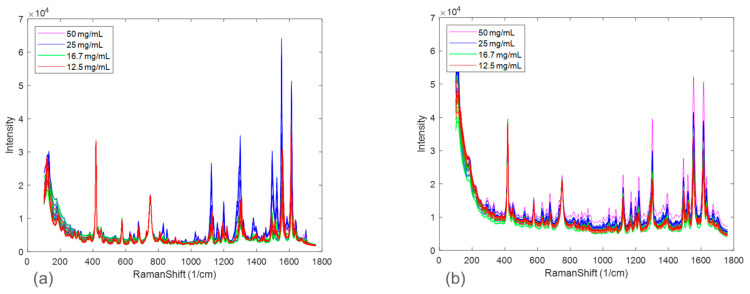
Calibration spectra for HCl salt (**a**) and maleate salt (**b**).

**Figure 6:**
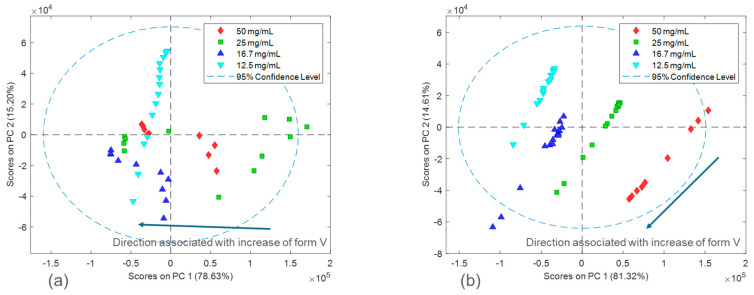
PCA scores of the calibration sets for HCl salt (**a**) and maleate salt (**b**).

**Figure 7:**
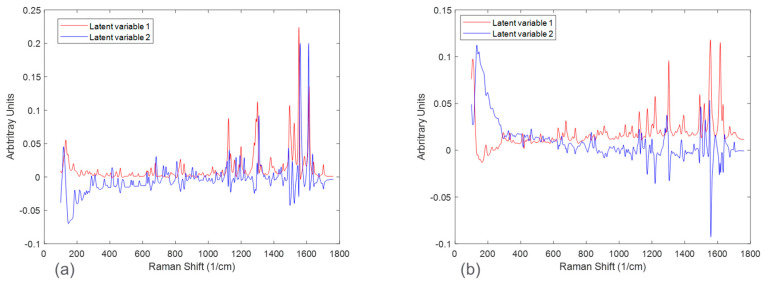
Latent variable plots of the calibration sets for HCl salt (**a**) and maleate salt (**b**).

The spectral differences between the experimental runs of the two salts were further investigated by performing principal component analysis (PCA). Figure 5 shows the calibration Raman spectra used in the PCA analysis. The results of the PCA analysis are shown in Figure 6 and Figure 7 in the form of scores plots and latent variable plots, respectively. For the HCl salt, the scores plot showed each experimental run clustered together and moving towards the same region in the PCA space. The movement in the left direction corresponds to an increase in the stable polymorph form V fraction. In addition to polymorph ratio changes, PC 1 and PC 2 also include variability associated with potential interferences such as solid concentration and interaction effects. According to the corresponding latent variable plot (Figure 7a), the shapes corresponding to PC 1 and PC 2 are primarily due to peak height changes. This matches the conclusion from Figure 3, which suggested that the primary spectral change from solid concentration for HCl salt was peak height variations.

A PCA analysis for the maleate salt runs revealed a different variance structure compared to the HCl salt runs. The scores plot showed that the trend for each solid concentration was similar; however, they did not overlap in PC space. The direction of the polymorph ratio was towards the bottom left; thus, both PC 1 and PC 2 were needed to explain variance due to form changes. The corresponding latent variable 1 plot showed that the shape associated with the most variability is due to peak height changes. The latent variable 2 plot consistently showed a peak increase followed immediately by a peak decrease at several positions, especially near the Raman shift of 1300 cm^−1^. This was indicative of peak shifts, which have greatly contributed to the overall variability. Again, this matches the conclusion from Figure 3, in which solid concentration changes were a function of both peak height changes and peak shifts. In general, for both salts, there were signal variations associated with polymorph ratio in the Raman calibration sets allowing for quantitative modeling. Potential interferences associated with solid concentration and interactions required chemometric techniques to mitigate their effects. These chemometric techniques had to be further optimized for each salt condition to account for their differences in variance structure.

### 3.2. Baseline Models

**Figure 8:**
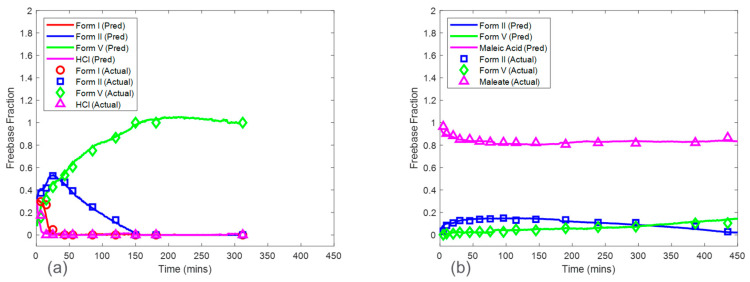
Baseline model predictions on test run condition of 16.7 mg/mL for the HCl salt experiment (**a**) and the maleate salt experiment (**b**). Note the relative standard deviation of XRPD reference values was approximately 4%.

Baseline model predictions were shown in Figure 8 for both the HCl salt experiment and the maleate salt experiment. The model accuracy was evaluated using RMSECV. The RMSECVs for the three baseline models corresponding to the HCl salt experiment were 0.056, 0.034, and 0.022 mass fractions of form I, form II, and form V, respectively. For the maleate salt experiment, the RMSECVs for the two baseline models were 0.016 and 0.023 mass fractions of form II and form V, respectively. According to these results, the form II model had an approximately two times higher RMSECV in the HCl salt experiment when compared to the maleate salt experiment. This was potentially due to the observed form I present in only the HCl salt experiment, which added another source of variance in the Raman dataset. See Section 3.5 for further model comparison.

### 3.3. Models from Calibration Procedure without Inclusion of Concentration Effect

**Figure 9:**
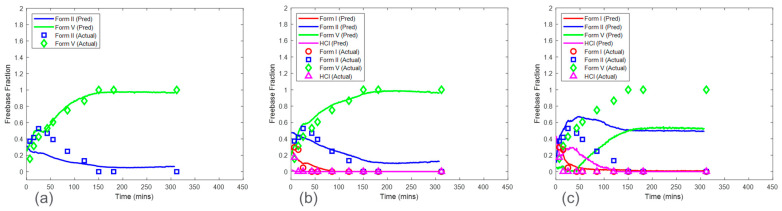
Model prediction on HCl salt experiments using the calibration without concentration procedure: 50 mg/mL (model set 1) (**a**), 25 mg/mL (model set 2) (**b**), and 12.5 mg/mL (model set 3) (**c**) solid concentrations. Note the relative standard deviation of XRPD reference values was approximately 4%.

The CP was used to further evaluate the effect of solid concentration in model development and whether the PLS algorithm could mitigate the effect of this interference. Figure 9 shows the predictions of HCl salt disproportionation from model sets 1–3 (see Section 2.6.1). Overall, model set 2, which was associated with a calibration run condition of 25 mg/mL solid concentration, was the preferred choice when considering all the factors. The RMSEPs for model set 2 were 0.056, 0.094, and 0.070 mass fractions for form I, form II, and form V, respectively. Model set 1 included the most accurate model for form V with an RMSEP of 0.061 mass fraction. However, the prediction error for form II was the highest, with an RMSEP of 0.161 mass fraction. Note that there were no predictions shown for form I in model set 1 since form I was not present in the calibration run condition of 50 mg/mL. This highlights an important point to consider using this calibration procedure. All the polymorphs at various ratios were required to be present in the calibration dataset to capture the in situ Raman spectral variance needed for accurate and robust model development. As this was not the case with model set 1, these models would not be recommended. In contrast, the calibration run (solid concentration of 12.5 mg/mL) for model set 3 included all the observed polymorphs. However, model set 3 suffered from severe model bias resulting in high prediction error for form II (RMSEP of 0.345 mass fraction) and form V (RMSEP of 0.443 mass fraction). These results supported the initial PCA analysis, which showed that the variance structure for the run condition of 12.5 mg/mL was unique when compared to the other solid concentrations (Figure 6). In this case, the PLS algorithm coupled with mean-centered preprocessing was not able to successfully use the spectral variance from the solid concentration of 12.5 mg/mL to create a model for predicting the test run with a solid concentration of 16.7 mg/mL. This could be because of the effects of suspension density on Raman spectra [24]. A decrease in suspension density reduces the signal-to-noise ratio. This is due to the linear relationship of the density of the scattering material with the Raman signal intensity at the site of spectral collection [25]. The higher suspension density corresponding to higher solid concentration could be enhancing the signal-to-noise ratio. In general, model set 2 was the preferred option but still had a high error of 0.094 mass fraction for the form II model. These results suggested that solid concentration effects needed to be considered in model development for the HCl salt experiments; therefore, the CP_Conc_ was also investigated in Section 3.4.

**Figure 10:**
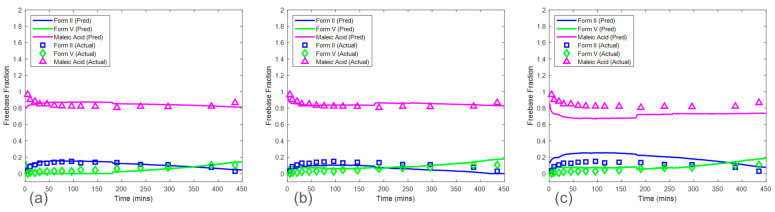
Model prediction on maleate salt experiments using the calibration without concentration procedure: 50 mg/mL (model set 1) (**a**), 25 mg/mL (model set 2) (**b**), and 12.5 mg/mL (model set 3) (**c**) solid concentrations. Note the relative standard deviation of XRPD reference values was approximately 4%.

Similarly, model predictions for model sets 1–3 were generated for the maleate salt experiments (Figure 10). This set of models included only form II and form V models, as form I was not present in any of the maleate salt experiments. The prediction error for form V was consistent across the three model sets ranging from 0.043 to 0.057 mass fraction of form V. In contrast, form II RMSEP increased from 0.018 mass fraction in model set 1 to 0.094 mass fraction in model set 3. The RMSEP approximately doubled as the calibration run condition decreased in solid concentration by a factor of two. Therefore, similar to the HCl salt experiments, model set 3 had the highest error and bias for form II, which propagated to high bias in maleate salt mass fraction calculation. These results again suggest that solid concentration has a strong effect on model performance and that at the low solid concentration, the reduction of the Raman signal associated with the polymorphs was reducing the model accuracy. This phenomenon suggested that the calibration runs with a solid concentration of 12.5 mg/mL were below a suspension density threshold and associated with a low signal-to-noise ratio for both salt experiments [24]. The prediction performance of model sets 1 and 2 were much better in maleate salt experiments than in HCl salt experiments, suggesting that at higher solid concentrations, the PLS algorithm was able to mitigate the effect of interferences and produced accurate models in the maleate salt experiments. The improved model performance at a higher concentration for the maleate salt experiments can be explained by the lower conversion rate of the salt, thus limiting the mass fraction range, and fewer sources of variance due to the presence of only two polymorphs.

### 3.4. Models from Calibration Procedure with Inclusion of Concentration Effect

**Figure 11:**
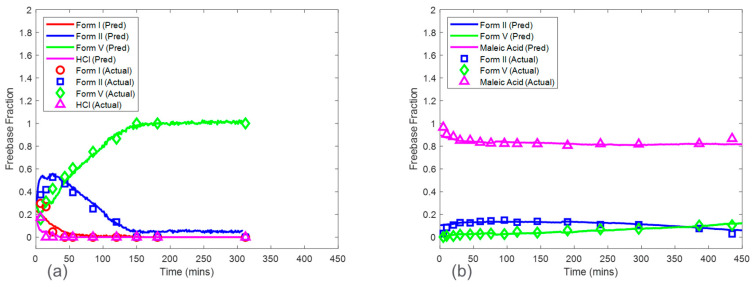
Model predictions of HCl salt (**a**) and maleate salt (**b**) disproportionation from concentration robust modeling procedure for 16.7 mg/mL concentration. Note the relative standard deviation of XRPD reference values was approximately 4%.

The predictions from the CP_Conc_ for the HCl salt experiments are shown in Figure 11a. The RMSEPs for the form I, form II, and form V models were 0.048, 0.056, and 0.065 mass fractions, respectively. These prediction errors were comparable to the cross-validation errors from the baseline model except for the form V model, which had an RMSEP approximately three times higher than the baseline model RMSECV. In addition, the RMSEP for the models from this procedure was generally lower than the RMSEP for the models generated from the CP. The high accuracy from the models based on the CP_Conc_ was due to a larger calibration Raman dataset, which included more variations of polymorph ratio. Interference from solid concentration was present in the calibration dataset and was addressed by performing SNV and autoscale preprocessing to reduce further effects of baseline and to normalize the peak height intensities. In addition, up to six latent variables were used to ensure that most of the variance associated with polymorph ratio changes was captured by the model. This modeling strategy produced both accurate and robust models for HCl salt experiments.

The CP_Conc_ also produced accurate models for maleate salt experiments (Figure 11b). The RMSEP for form II and form V models were 0.028 and 0.017 mass fractions, which were similar to the baseline RMSECV results. Additional spectral preprocessing was not required to produce accurate models, suggesting a reduced effect of solid concentration on the Raman dataset, with spectral variations from polymorph ratio being more dominant when compared to HCl salt experiments.

The CP_Conc_ was shown to produce accurate models for both HCl salt and maleate salt experiments in addition to being robust against solid concentrations in the range of 12.5 mg/mL to 50 mg/mL. The model predictions from this procedure were used in a subsequent section (Section 3.6) to discuss the kinetics of salt disproportionation.

### 3.5. Comparison between Calibration Procedures

The model results for both the HCl salt and maleate salt experiments are listed in Table 3 and Table 4, which show the RMSEPs for models generated with both the CP and CP_Conc_ strategies. For the baseline models, the results listed were RMSECV values since the calibration, and test experimental runs were the same. For the HCl salt experiments, form I had the highest RMSECV amongst the other baseline models. In addition, while not the highest in the model sets, form I model RMSEPs from both the CP and CP_conc_ strategies were fairly consistent with the form I RMSECV from the baseline model. These two points suggest that the form I spectral signal may not have been well defined in calibration datasets. Form I conversion was the fastest, leading to collecting as low as three reference samples in between 0 and 1 mass fraction for the calibration datasets. This implies that more polymorph ratios with form I variation would be needed to be able to build more accurate models for the prediction of form I mass fraction. The physical instability of form I may also have led to misleading XRPD reference values during offline measurements adding additional errors to form I models. Form II was the most difficult to predict as form II models generally had the highest RMSEPs amongst all the HCl salt models. The models produced with the CP corresponding to model sets 1 to 3 had models with RMSEP of 0.094 mass fraction or above. The CP_Conc_ was able to improve the model performance due to the combination of data from the three solid concentration calibration runs.

The maleate salt experiments had more accurate form II models when comparing models across the two salts in the CP when solid concentration for the calibration run was high, e.g., 25 mg/mL and 50 mg/mL. The form II model for the CP of maleate salts at 50 mg/mL concentration was more accurate than the CP_Conc_ form II model. When collectively considering the RMSEP from form I and form II models, the CP_Conc_ models were the most accurate for maleate salt experiments.

Pharmaceutical scientists may select the calibration procedure based on the required accuracy of the model and the resources available to perform the experimental runs. The advantage of the baseline and CP-based models was that only one experimental run was needed, thus reducing the resource requirement associated with producing several controlled mixtures for calibration according to a traditional DoE. If additional interferences needed to be considered, such as solid concentration, additional controlled experimental runs can be performed to facilitate the creation of robust and accurate models, e.g., the CP_Conc_. However, the proposed calibration procedures required prior consideration of three assumptions. The first assumption was that all the polymorphs had an associated Raman signal, which could be deconvoluted. The second assumption was that all relevant polymorphs needed to be present, and their ratio variation was captured with the in-line Raman sensor during the calibration disproportionation runs. This assumption was not met for the HCl salt model set 1 since one of the relevant polymorphs (form I) was not present in the calibration run. This was due to the physical instability of form I, which rapidly converted to other polymorphs, causing difficulty in capturing Raman spectral variation associated with form I mass fraction. The third assumption was that the reference XRPD measurements were considered to provide the actual mass fractions. The quantitative Raman models rely on the reference values for development and evaluation. Therefore, the XRPD method needed to be able to differentiate the polymorphs, and the polymorph samples needed to be stable throughout the XRPD measurement to deliver accurate measurements. In conclusion, the selection of the calibration procedure for assessing the disproportionation of a salt was based on the Raman sensitivity to the polymorph ratio, the presence of all relevant polymorphs during disproportionation experiments, and the ability to obtain accurate reference X-ray measurements.

### 3.6. Model Application for Salt Selection

The Raman in situ quantitative models were developed to understand the kinetics of salt disproportionation. The following kinetic discussion was based on model set 4 from CP_conc_ due to its relatively higher accuracy and robustness. For both the HCl and maleate acid salt test runs, the parent salt mass fraction at the beginning was approximately 1. At the end of the test run experiments, the salt mass fraction was approximately 0 for HCl and 0.81 for maleate acid salt, respectively. Therefore, the HCl salt showed a complete disproportionation, in which the end-product was approximately 100% of the thermodynamically most stable freebase polymorph. The maleate acid salt showed partial disproportionation, in which the end-product was approximately 81% maleate acid salt, 6% form II, and 13% form V in mass. Assuming zero-order kinetics, HCl salt converted at a faster rate of approximately 14% per minute when compared to maleate salt conversion rate of approximately 0.01% per minute. These results clearly showed that maleate salt was physically more stable than HCl salt even when having comparable solubilities. It is important for the salt to be stable while in the gastrointestinal tract, where pH swings are prevalent. The longer the salt form is maintained in these gastrointestinal conditions, the longer the solubility advantage exists for the drug, thus, enhancing dissolution and overall drug exposure. In this case, the maleate salt was chosen over the HCl salt as the drug substance for further development. Future work will involve a complete mechanistic assessment of the disproportionation pathways for maleate salt facilitated by the in situ Raman models developed in this study.

## 4. Conclusions

In this work, the disproportionation of two salts and their specific polymorphs was monitored with in situ Raman spectroscopy and offline X-ray diffraction measurements. Quantitative models were developed to track the amount of each polymorph during experimental runs. The existence of up to four solid forms (salt and three freebase forms) presented a tremendous challenge in creating controlled samples for model development. Therefore, two calibration procedures without and with concentration effect (CP and CP_Conc_) were developed, tested for their robustness against solid concentrations, and used to guide the selection of the salt.

Three model sets were created using CP for each salt. Each model set was based on one calibration run experiment with a particular starting solid concentration and evaluated using a test run experiment with a different solid concentration. In this way, the effect of solid concentration on model performance was demonstrated. This procedure was used for two salts: HCl salt and maleate salt. The models generated for the maleate salt generally had better prediction performance with lower RMSEPs when compared to the HCl models. The models for both salts showed that the prediction errors were impacted by solid concentrations in that at lower solid concentrations, the prediction errors (RMSEPs) were higher.

The other calibration procedure investigated in this study was the CP_Conc_. This procedure involved a calibration dataset encompassing the solid concentration variations from three experimental runs. The models for the HCl salt and maleate salt experiments from this procedure overcame accuracy concerns when applying to different solid concentrations in that lower prediction errors (RMSEPs) were obtained when compared to the models from the CP. These models were used for assessing the physical stability of the salts via understanding the kinetics of disproportionation. Maleate salt was significantly more stable than HCl salt, with a conversation rate of approximately 0.01% per minute compared to 14% per minute for HCl salt. Therefore, maleate salt was selected as the drug substance for further development. This work introduced two calibration procedures to enable quantitative in situ Raman monitoring of disproportionation complicated by more than two polymorphs at different solid concentrations.

## Figures and Tables

**Table 1:** The set of four disproportionation runs for each salt and their corresponding starting solid concentration.

	Disproportionation Runs for HCl or Maleate Salt			
	Calibration Run 1	Calibration Run 2	Calibration Run 3	Test Run
Solid Concentration (mg/mL)	50.0	25.0	12.5	16.7
Raman Data Collection	Every other minute	Every other minute	Every other minute	Every other minute
XRPD Data Collection	9 measurements	12–17 measurements	15 measurements	10–15 measurements

**Table 2:** Four sets of models for calibration procedure without and with the inclusion of concentration effect.

Calibration Procedure	Model Set	Calibration/Test Run for Model Development:
Baseline	Baseline Models	Test Run
CP	Model Set 1	Calibration Run 1
	Model Set 2	Calibration Run 2
	Model Set 3	Calibration Run 3
CP_conc_	Model Set 4	Calibration Run 1 + 2 + 3

**Table 3:** Prediction performance of each model generated for the maleate acid salt experiments. Note that the % relative standard deviation of Raman quantitation was approximately 1.12%, and all models had R^2^ > 0.88.

Maleate Salt RMSEP (Mass Fraction) Results								
Calibration Procedure	Model Set	Calibration Run Condition (mg/mL)	Test Run Condition (mg/mL)	Form I (RMSEP)	Form II (RMSEP)	Form V (RMSEP)	Preprocessing	Number of Latent Variables
Baseline	Baseline Models	N/A	16.7	N/A	0.016 *	0.023 *	Mean center	N/A, 2, 3
CP	Model Set 1	50.0	16.7	N/A	0.018	0.057	Mean center	N/A, 4, 2
	Model Set 2	25.0	16.7	N/A	0.044	0.044	Mean center	N/A, 3, 3
	Model Set 3	12.5	16.7	N/A	0.094	0.043	Mean center	N/A, 1, 1
CP_conc_	Model Set 4	50 + 25 + 12.5	16.7	N/A	0.028	0.017	Mean center	N/A, 2, 4

* RMSECV Provided for Baseline Models.

**Table 4:** Prediction performance of each model generated for the HCl salt experiments. Note that the % relative standard deviation of Raman quantitation was approximately 1.12%, and all models had R^2^ > 0.88.

HCl Salt RMSEP (Mass Fraction) Results								
Calibration Procedure	Model Set	Calibration Run Condition (mg/mL)	Test Run Condition (mg/mL)	Form I (RMSEP)	Form II (RMSEP)	Form V (RMSEP)	Preprocessing	Number of Latent Variables
Baseline	Baseline Models	N/A	16.7	0.056 *	0.034 *	0.022 *	Mean center	4, 4, 3
CP	Model Set 1	50.0	16.7	N/A	0.161	0.061	Mean center	N/A, 3, 2
	Model Set 2	25.0	16.7	0.056	0.094	0.070	Mean center	2, 4, 3
	Model Set 3	12.5	16.7	0.045	0.345	0.443	Mean center	5, 3, 4
CP_conc_	Model Set 4	50 + 25 + 12.5	16.7	0.048	0.056	0.065	SNV/Auto	6, 5, 5

* RMSECV Provided for Baseline Models.

## Data Availability

Data is contained within the article.

## References

[1] Babu N.J., Nangia A. (2011). Solubility advantage of amorphous drugs and pharmaceutical cocrystals. Cryst. Growth Des..

[2] Gould P.L. (1986). Salt selection for basic drugs. Int. J. Pharm..

[3] Serajuddin A.T. (2007). Salt formation to improve drug solubility. Adv. Drug Deliv. Rev..

[4] Berge S.M., Bighley L.D., Monkhouse D.C. (1977). Pharmaceutical salts. J. Pharm. Sci..

[5] Avdeef A., Sugano K. (2022). Salt Solubility and Disproportionation–Uses and Limitations of Equations for pHmax and the In-silico Prediction of pHmax. J. Pharm. Sci..

[6] Elder D.P., Holm R., De Diego H.L. (2013). Use of pharmaceutical salts and cocrystals to address the issue of poor solubility. Int. J. Pharm..

[7] Hsieh Y.-L., Merritt J.M., Yu W., Taylor L.S. (2015). Salt stability–the effect of pHmax on salt to free base conversion. Pharm. Res..

[8] Patel M.A., Luthra S., Shamblin S.L., Arora K.K., Krzyzaniak J.F., Taylor L.S. (2018). Effect of excipient properties, water activity, and water content on the disproportionation of a pharmaceutical salt. Int. J. Pharm..

[9] Gupta D., Bhatia D., Dave V., Sutariya V., Varghese Gupta S. (2018). Salts of therapeutic agents: Chemical, physicochemical, and biological considerations. Molecules.

[10] Unger E.F. (2009). Weighing benefits and risks—The FDA’s review of prasugrel. N. Engl. J. Med..

[11] Hsieh Y.-L., Yu W., Xiang Y., Pan W., Waterman K.C., Shalaev E.Y., Shamblin S.L., Taylor L.S. (2014). Impact of sertraline salt form on the oxidative stability in powder blends. Int. J. Pharm..

[12] Box K., Comer J. (2008). Using measured pKa, LogP and solubility to investigate supersaturation and predict BCS class. Curr. Drug Metab..

[13] Food and Drug Administration (2004). Guidance for Industry, PAT-A Framework for Innovative Pharmaceutical Development, Manufacturing and Quality Assurance. http://www.fda.gov/cder/guidance/published.html.

[14] Nie H., Liu Z., Marks B.C., Taylor L.S., Byrn S.R., Marsac P.J. (2016). Analytical approaches to investigate salt disproportionation in tablet matrices by Raman spectroscopy and Raman mapping. J. Pharm. Biomed. Anal..

[15] Wray P.S., Sinclair W.E., Jones J.W., Clarke G.S., Both D. (2015). The use of in situ near infrared imaging and Raman mapping to study the disproportionation of a drug HCl salt during dissolution. Int. J. Pharm..

[16] Ewing A.V., Wray P.S., Clarke G.S., Kazarian S.G. (2015). Evaluating drug delivery with salt formation: Drug disproportionation studied in situ by ATR-FTIR imaging and Raman mapping. J. Pharm. Biomed. Anal..

[17] Nie H., Klinzing G., Xu W. (2022). A comparative study of applying backscattering and transmission Raman spectroscopy to quantify solid-state form conversion in pharmaceutical tablets. Int. J. Pharm..

[18] Lenain B., Lucas H., Uerpmann C., Davis K.L., Ehly M.A., Kemper M.S., Lewis I.R. (2007). Raman spectroscopy for process control in chemical and pharmaceutical manufacturing. STP Pharma Tech. Prat. Reglem..

[19] Paudel A., Raijada D., Rantanen J. (2015). Raman spectroscopy in pharmaceutical product design. Adv. Drug Deliv. Rev..

[20] Fung P., Mah H., Goldberg A.F., Rieder C., Sun H.-Y., Su Z., Du J., Lai C. (2020). PAT-Facilitated Pharmaceutical Crystallization Development through Mechanistic Understanding. Cryst. Growth Des..

[21] Xu Z., He Z., Song Y., Fu X., Rommel M., Luo X., Hartmaier A., Zhang J., Fang F. (2018). Topic review: Application of Raman spectroscopy characterization in micro/nano-machining. Micromachines.

[22] Simone E., Saleemi A.N., Nagy Z. (2014). Application of quantitative Raman spectroscopy for the monitoring of polymorphic transformation in crystallization processes using a good calibration practice procedure. Chem. Eng. Res. Des..

[23] Chen Z.-P., Fevotte G., Caillet A., Littlejohn D., Morris J. (2008). Advanced calibration strategy for in situ quantitative monitoring of phase transition processes in suspensions using FT-Raman spectroscopy. Anal. Chem..

[24] Helmdach L., Feth M.P., Ulrich J. (2013). Integration of process analytical technology tools in pilot-plant setups for the real-time monitoring of crystallizations and phase transitions. Org. Process Res. Dev..

[25] Cornel J., Lindenberg C., Mazzotti M. (2008). Quantitative application of in situ ATR-FTIR and Raman spectroscopy in crystallization processes. Ind. Eng. Chem. Res..

